# Age and gender distribution of firearm violence in high-income countries: an analysis of data from 1990 to 2019

**Published:** 2025-07

**Authors:** Moslem Taheri Soodejani, Marzieh Mahmudimanesh, Marjan Rasoulian-Kasrineh, Seyed Jalaleddin Mousavirad, Seyyed Mohammad Tabatabaei

**Affiliations:** ^*a*^ Center for Healthcare Data Modeling, Departments of biostatistics and Epidemiology, School of Public Health, Shahid Sadoughi University of Medical Sciences, Yazd, Iran.; ^*b*^ Department of Public Health, Esfarayen Faculty of Medical Science, Esfarayen, Iran.; ^*c*^ Department of Medical Informatics, School of Medicine, Mashhad University of Medical Sciences, Mashhad, Iran.; ^*d*^ Department of Computer and Electrical Engineering, Mid Sweden University, Sundsvall, Sweden.; ^*e*^ Clinical Research Unit, Imam Reza Hospital, School of Medicine, Mashhad University of Medical Sciences, Mashhad, Iran.

**Keywords:** Gun violence, Physical violence, Firearms, Incidence, Clustering

## Abstract

**Background:**

Physical Violence by Firearms (PVF) is a type of violence which is considered a public health challenge in high-income countries. This study is designed to investigate the trend of incidence in these countries among different ages and gender groups, cluster countries based on PVF incidence rates, and analyze changes during the years 1990 to 2019.

**Methods:**

At first, countries were clustered using the K-means algorithm, with the number of clusters deter-mined by the elbow method. The clustering was based on the Euclidean distance of physical vio-lence by Firearms (PVF) incidence rates, and the data were sourced from the Global Burden of Disease (GBD) database. The annual changes in the incidence in each cluster were calculated by means of sex and age groups. A heat map was also used to investigate the trend of firearms violence, and Arc map GIS was employed to provide the geographical incidence distribution of firearms violence by gender in 4-time points of 1990, 2000, 2010 and 2019.

**Results:**

The United States, which was placed alone in a cluster, had the highest incidence changes with an increase of 1.44 cases per 100,000 per year. The highest incidence of violence was among American men aged 20-24, which ranged from 150 to 240 cases per 100,000 people between 1990 and 2019.

**Conclusions:**

The study highlights that access to firearms and related laws are key drivers of the increasing trend of PVF in high-income countries. The clustering of countries revealed distinct patterns of PVF incidence, with the USA showing the highest rates. These findings underscore the need for stricter firearm regulations and targeted interventions, particularly for young men aged 20-24, who are most affected by PVF.

## Introduction

Violence is a public health problem that has recently increased at all social levels. As a necessary type, Physical Violence by Firearms (PVF) occurs in different countries, and has become a serious challenge to the health of other people in society, especially young women.^1,2^

As a high-income country, the United States of America (USA) is severely dealing with this challenge, so in 2011, more than 32,000 deaths were recorded as being caused by PVF.

Although it might be underreported, it reached nearly 48,000 deaths in 2021, indicating an increase in the trend of (PVF) in this country.^3^ Overall, 40% of all PVF happen as homicide and 50% as suicide.^4^

In addition to the USA, other high-income countries also deal with PVF, which can impose high financial, social, and psychological costs on them. For example, Sweden also confirmed the increase in PVF, which may cause this country to suffer from damages such as an increase in suicides, a decrease in citizen acceptance, and an increase in the need for medical services in the future.^5^

Violence causes irreparable damage at all ages, but it might be worse at younger generations. In children, these injuries can be much more severe. In 2016, PVF was the third leading cause of death in children under the age of 19, which included about 14%.^6^ Even if it does not lead to death, it can cause irreparable damage and disorders such as post-traumatic stress disorder (PTSD), anxiety, and depression. On the other hand, it may increase the risk of suicide among teenagers.^7^

Women are other severe victims of this type of violence in developing or low-income countries; for example, the risk of homicide has increased in South Africa due to the widespread use of weapons, which has made women the primary homicide victims killed by their partners.^8^ This difference may also exist in high-income countries, which this study seeks to find out.

As a high-income country, the USA reports the highest rates of gun violence, but is this rate increasing? What is the trend in other high-income countries? How is this trend in different genders and age groups? How has this rate changed in high-income countries between 1990 and 2019? These are the questions that will be answered in this study.

To the best of our knowledge, no research with these goals, questions and title has been conducted in high-income areas investigating the trend of PVF among each age and gender group before. Therefore, it can be said that this study is very important and useful for policy makers because they have the right to make decisions to deal with this challenge.

**Literature Review**

Also, to complete previous information related to this topic, the results of articles related to keywords "Firearms", "Gun", "violence", and "Incidence" were reviewed in PubMed and Google Scholar. Ten articles were found in PubMed and 22 articles in Google Scholar that contained these keywords in their titles or abstracts. Most of these studies investigated the use of firearms and their laws in the United States of America. The results of the article by Saadi et al. have shown that the use of firearms for suicide is more common among elderly.^9^ It is more frequent in Florida, which also has a larger elderly population.^10^

The high frequency of PVF in high-income countries, especially the United States of America, can be related to many reasons. In a study by Fingerhut et al. has shown that usually certain groups of children and especially adolescents such as young black boys and teenagers are at risk of death by firearms. Firearm injuries account for far more deaths than other injuries for which children and youth visit emergency departments reflecting the extreme lethality of firearms. ^11^ However, some laws and health approaches have been put in place to prevent PVF from happening at schools and among other age groups, which may help reduce injuries and deaths from it.^12,13^

## Methods 

**GBD database**

The GBD study is a comprehensive regional and global burden of a disease research program that assesses mortality and disability from major diseases, injuries, and risk factors. To conduct such research, updated data on world health status from 1990 to 2019 by age and sex groups in different geographic regions are available on the website developed by the Institute for Health Measurement and Evaluation (IHME). The data used in this study, which focuses on the occurrence of unintentional violence per 100,000 people and its trend from 1990 to 2019 in high-income countries by different age groups of men and women, were extracted from the Global Burden of Disease website (https://ghdx.healthdata.org/). In these variables, high-income countries are countries that have a higher Gross National Income (GNI) per capita than the others. Based on geographical regions, they are generally divided into five categories: Australasia, High-income Asia Pacific, Southern Latin America, Western Europe, and high-income North America.

**K-means clustering**

High-income countries were clustered using the K-means algorithm, into four categories by chosen for its efficiency in handling large datasets and its ability to minimize within-cluster variance. The optimal number of clusters (four) was determined using the elbow method, which evaluates the reduction in within-cluster sum of squares (WCSS) as the number of clusters increases. The four countries with the most considerable distance in terms of incidence were selected as the cluster head, and based on the Euclidean distance, the countries with the smallest distance from the cluster head in terms of PVF rate were placed in these 4 clusters. The initial cluster centroids were selected through an iterative max-min process to ensure maximal dispersion. we computed pairwise Euclidean distances between all countries. The first centroid was the country with highest average distance to others; subsequent centroids were selected as countries maximizing the minimum distance to already-chosen centroids. This approach, combined with 100 random restarts, helped mitigate initialization sensitivity. Finally, there are countries that are most similar to each other in terms of PVF incidence in each cluster while having the largest difference with the countries in other clusters.

**Heat map Chart**

Heat map plots were used to track the changes in PVF incidence in different age groups among men and women. For this purpose, nine age groups were created including less than 20 years, 20 to 24 years, 25 to 29 years, 30 to 34 years, 35 to 39 years, 40 to 44 years, 45 to 49 years, 50 to 69 years and more than 70 years. The changes in each age group were characterized by a color range from dark green to red (low to high incidence). These graphs are provided for all high-income regions, separately.

**Geographical Distribution**

The geographic distribution of PVF incidence in high-income countries was investigated using ArcMap GIS version 10.2; to do that, the countries were evaluated separately for men and women during four periods. The incidence statuses of the countries were colored according to the cut-point of the quartiles (Q1, Q2, Q3, and Q4), where Q1 represents the lowest 25% of PVF incidence rates, Q2 the next 25%, and so on.

## Results

The clustering of PVF incidence showed that the USA was placed in a separate cluster, and Malta and Chile were placed in another cluster alone. Although the annual incidence trend in different clusters demonstrates an increase in PVF incidence in all these 4 clusters, the most significant changes were related to the men in cluster 3 (Malta, Chile), which has an annual average increase of 1.14 PVF per hundred thousand people, which was 0.44 for the USA. The yearly incidence changes among women were not noticeable, and the biggest annual changes per hundred thousand people have belonged to the USA, which was reported as 0.07. Overall, the most changes were related to the USA with 0.25 among both sexes (Table 1).

**Table 1:** Year to year change of firearm incidence by gender and cluster. Coefficients represent Annual Percent Change (APC).

Gender	Clusters	Coefficient	Standard Error	P
**Male**	Cluster1	0.08	0.01	<0.01
	Cluster2	0.05	0.17	<0.01
	Cluster3	1.14	0.24	<0.01
	Cluster4	0.44	0.12	<0.01
**Female**	Cluster1	0.02	0.01	<0.01
	Cluster2	0.02	0.02	0.3
	Cluster3	0.07	0.02	<0.01
	Cluster4	0.01	0.02	0.9
**Both**	Cluster1	0.05	0.01	<0.01
	Cluster2	0.25	0.07	<0.01
	Cluster3	0.23	0.08	<0.01
	Cluster4	0.01	0.01	0.9

Cluster1: Brunei Darussalam, Japan, Republic of Korea, Singapore, Australasia New Zealand, Andorra, Austria, Belgium, Cyprus, Denmark, Finland, France, Germany, Greece, Iceland, Ireland, Italy, Luxembourg, Netherlands, Norway, Portugal, Spain, Sweden, Switzerland, United Kingdom, Greenland, Monaco, San Marino. Cluster2: Israel, Argentina, Uruguay, Canada. Cluster3: United State of America. Cluster4: Malta, Chile.

The annual changes of PVF incidence in high-income countries, as well as Australia showed that the trend is almost constant among men and women. Besides, the age less than 20 years and over 70 years had the highest rate in both sex groups. In high-income Asian pacific, an increasing trend of PVF among both sexes and under 20 years of age.

In Southern Latin America, the trend is almost constant in women, but there is an increasing trend among men under the age of 20 and between 35 and 39. These changes have occurred among women between the ages of 20 and 24 and men between 40 and 44 in Western Europe.

**Figure 1:**
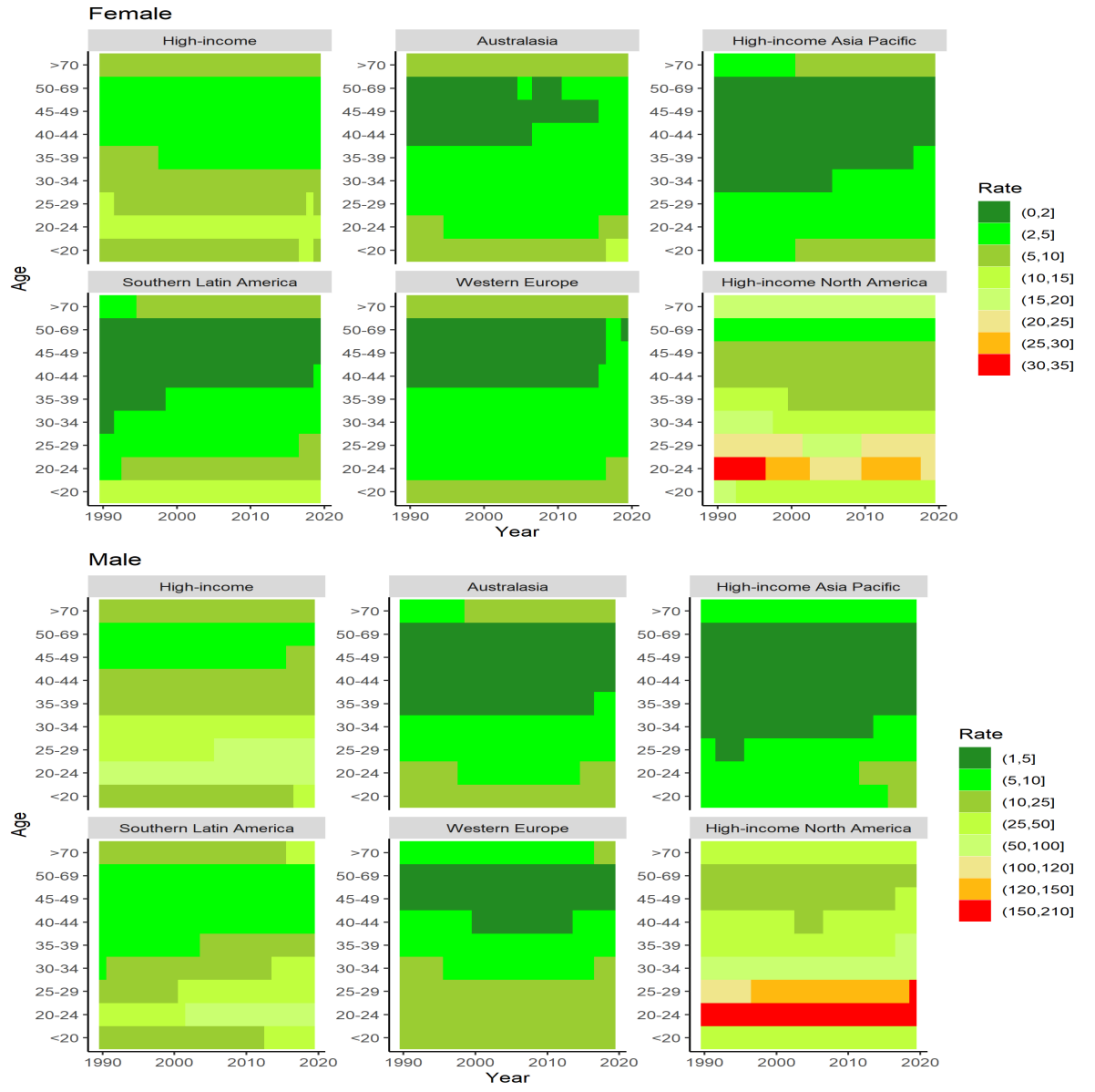
Heatmap Plot for Incidence Rate of Gun Violence in High-income Countries by Age Groups and Sex, 1990-2019

There were changes in the PVF incidence among the age group of 25 to 29, in high-income North America; however, it was higher in this region than the others among all age groups and both. Although the trend of PVF incidence in men aged 20 to 24 years in high-income North America is unchanged, it has the highest incidence among all age groups in all regions. More details are provided in Figure 1.

**Figure 2:**
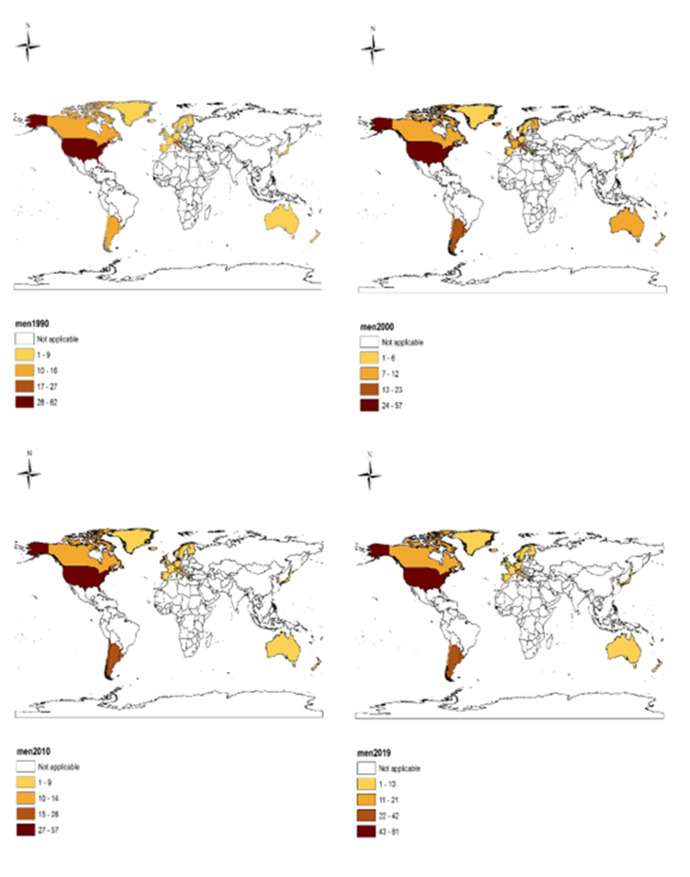
Geographical distribution of unintentional firearm violence in High income countries in four decades (men

**Figure 3:**
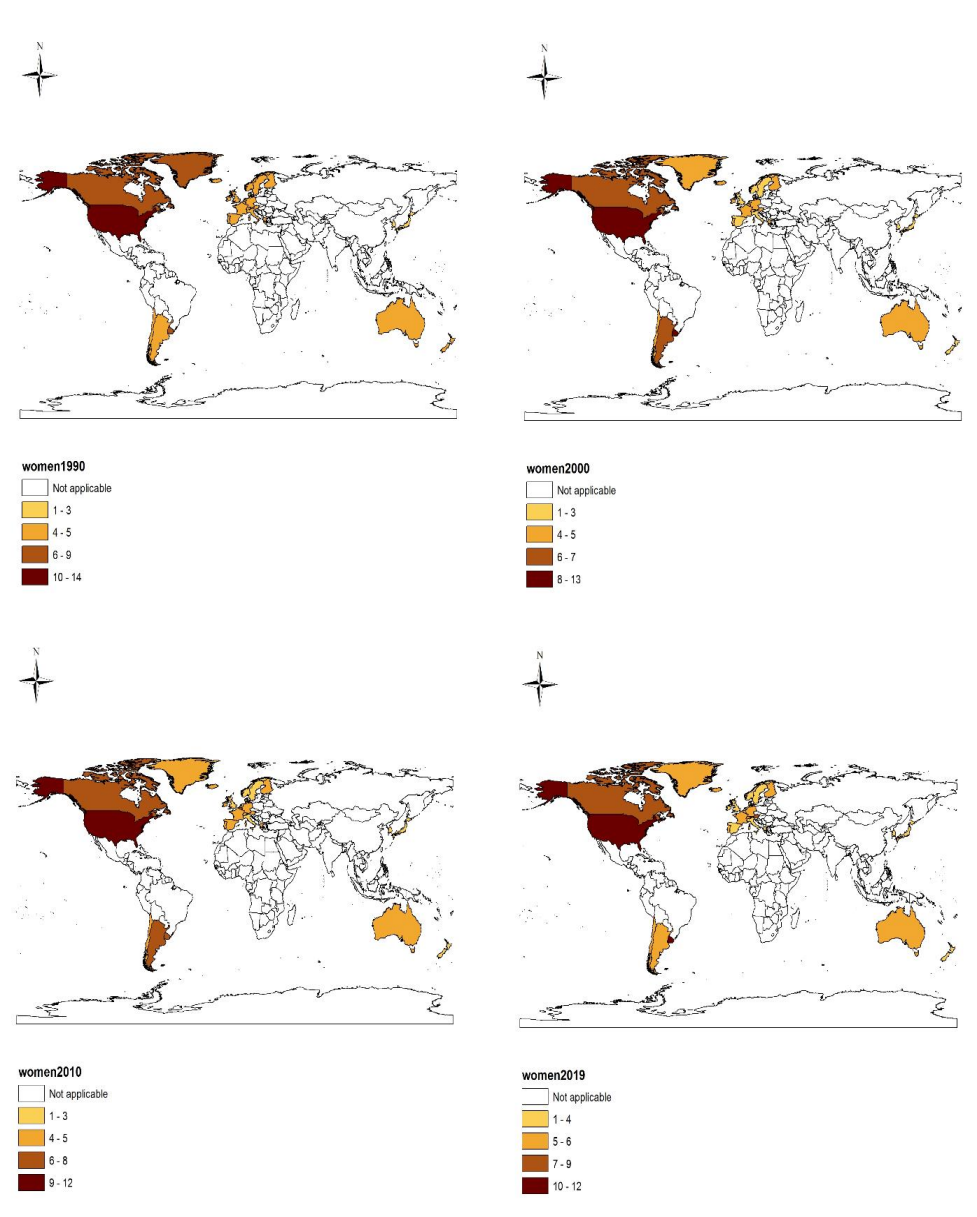
Geographical distribution of unintentional firearm violence in High income countries in four decades (women)

The geographic distribution of PVF incidence during four decades showed that high-income North America had the highest rate among other regions during this period and among both men and women. After this region, the southern America region had the highest PVF incidence during these four decades, which increased from 10-16 cases per 100,000 people in 1990 to 22-44 cases in 2019 (Figure 2,Figure 3).

## Discussion

The results of this study showed that during the years 1990 -2019, the high-income countries of North America had the highest incidence of PVF. This rate has always been higher among men than women in all age groups. The increase in this incidence in two countries, Malta and Chile, was higher than in others. It was even higher than in the USA.

In addition, the results of other studies showed that the USA had the highest incidence of PVF among all high-income countries, so the PVF rate in this country is 25 times that of other developed countries. 

According to the previous studies, the United States of America also ranks first in the rate of firearm deaths, and the rate has also increased from 2003 to 2015, while it has decreased in other high-income countries.^14^ In this article, it was also shown that North American countries have the highest rate of PVF. In older age, the most cases of firearm deaths were in men and due to suicide. The relatively higher rate of PVF in North America compared to other regions, in the age of 70 and above, can also be due to easier access to weapons and the high rate of suicide in the elderly of this region.^15^

The main reason for this high incidence might be the lack of restrictive laws about carrying a firearm in this country. Since it is not so challenging to access weapons in this country, this type of violence can be a serious threat to all people, especially those in the younger age groups. 

The high rate of PVF among youth in high-income countries in North America can be attributed to the high rate in the United States of America. On the other hand, the rate of homicide using firearms among young non-white males is the highest in this country. Also, firearm violence is related to income inequality and social capital. Therefore, easy access to weapons, racism in the culture of some regions, and social and income inequalities between whites and blacks can be some reasons for the high rate of firearm violence in North America, and specifically in the United States of America.^15,16^

There are various evidences proving that the access to weapons in the USA is increasing; On the other hand, many changes in the laws related to the carrying of firearms in different states of the USA between 1991 to 2016, have caused a change in the rate of PVF in this country, however in the other states where there was no strict firearm law, there was a higher PVF than the others.^17-19^

This kind of violence is seen most often in people between the ages of 15 and 24. Age is a significant factor when it comes to health. In this type of violence, there was a meaningful relationship between age groups and the number of PVF cases, so younger people were more likely to be affected. Uncontrolled emotions in these age groups, as well as easy access to firearms, may be two important reasons for the high incidence of PVF among this age group, according to a study conducted in 169 countries around the world.^12^ Alcoholic beverage consumption is another factor that can increase the probability of PVF among various people in the society, especially young ones. Various studies have found a link between PVF and the alcoholic beverages consumption. For example, a study showed that drunk people are responsible for 9 million out of 12 million PVFs, which is a high proportion.^20^

Women are known to be one of the victims of violence in the world, but the results of this study showed that the ratio of PVF among women is much lower than men in all high-income countries. Contrary to these results, a study in Africa showed that they are one of the primary victims of PVF,^8^ which might be due to various reasons such as their social role in different societies, the government's support and efforts on programs against PVF among women and also, gun ownership rules in these communities

In recent years, PVF rates have stayed almost the same in European countries with high incomes. This may be because these countries have programs to control PVF. For example, in Sweden, Finland, and the Netherlands, national organizations like the Council for Crime Prevention, the National Research Institute for Legal Policy, and the department of Criminology at Lebien University are studying the murders that are happening in these countries, and a database has been developed to review and monitor these data, which can be a great help in reducing PVF.^21^

The highest growth of PVF happened in Malta, Chile, even more than in the USA. This increase can affect the future of public health in these two countries. One of the reasons for this increase can be related to their demographic characteristics. Another reason might be the increase in the amount of immigration, which can affect the population diversity as well as the health level of a country; knowing that the amount of immigration in Chile has increased in recent years; However, perhaps the most important reason for the increase in PVF in Chile is the increasing production of weapons in this country, which has led to increased access to weapons for all people.^22^

In the end, it should be mentioned that there were some limitations in this study, such as the limited access to some search databases in Iran, which made it impossible to access many studies in the field of investigating the trend of PVF and its rate among each sex/age group. However, based on the available studies, it was tried to provide good information about the types of societies, cultures and existing laws related to accessing any firearm and its impact on PVF in high-income countries, especially the United States of America, which has the highest death rate and the injuries from it.

Among the high-income countries, the United States had the highest incidence of PVF, so after the clustering, the USA has placed alone in a cluster. The incidence was higher among men aged 20 to 24 than in other age groups. This difference was constant during the study, but the increasing rate of violence in Malta and Chile was higher than in other countries; it seems that access to firearms and related laws are some of the critical reasons causing this type of violence to increase.

The clustering of high-income countries based on PVF incidence rates revealed distinct patterns, with the USA forming a separate cluster due to its exceptionally high rates of firearm violence. Malta and Chile, grouped together in another cluster, showed a significant increase in PVF incidence over the study period. These findings suggest that factors such as firearm accessibility, demographic characteristics, and regional policies play a crucial role in shaping PVF trends. The clustering approach allowed us to identify countries with similar PVF profiles, which can inform targeted interventions and policy recommendations.

## Conclusion

The findings of this study, including the clustering of countries based on PVF incidence rates and the identification of vulnerable age and gender groups, can inform the development of targeted public health policies to reduce firearm violence.

**Acknowledgements**

We would like to thank Institute for Health Metrics and Evaluation (IHME) for making the data available to do such a research. Also, we would like to thank the Clinical Research Development Unit, Imam Reza Hospital, Mashhad University of Medical Sciences, for their assistance in this manuscript.

**Authors’ Contribution:**

M.T.S. and S.M.T. designed the study. M.M , M.R.K. and S.J.M. participated in data collection. M.T.S and M.M. analyzed and interpreted the data. M.T.S and S.M.T supervised all the work. M.T.S, M.M and M.R.K prepared the draft and S.M.T and S.J.M revised it. All authors re-viewed and approved the final version.
